# Increased whitening efficacy and reduced cytotoxicity are achieved by the chemical activation of a highly concentrated hydrogen peroxide bleaching gel

**DOI:** 10.1590/1678-7757-2018-0453

**Published:** 2019-07-25

**Authors:** Diana Gabriela SOARES, Natália MARCOMINI, Carla Caroline de Oliveira DUQUE, Ester Alves Ferreira BORDINI, Uxua Ortecho ZUTA, Fernanda Gonçalves BASSO, Josimeri HEBLING, Carlos Alberto de Souza COSTA

**Affiliations:** 1 Universidade de São Paulo Universidade de São Paulo Faculdade de Odontologia de Bauru Departamento de Dentística, Endodontia e Materiais Odontológicos Bauru São Paulo Brasil Universidade de São Paulo, Faculdade de Odontologia de Bauru, Departamento de Dentística, Endodontia e Materiais Odontológicos, Bauru, São Paulo, Brasil.; 2 Universidade Estadual Paulista Universidade Estadual Paulista Faculdade de Odontologia de Araraquara Departamento de Dentística Restauradora Araraquara São Paulo Brasil Universidade Estadual Paulista – UNESP, Faculdade de Odontologia de Araraquara, Departamento de Dentística Restauradora, Araraquara, São Paulo, Brasil.; 3 Universidade Estadual Paulista Universidade Estadual Paulista Faculdade de Odontologia de Araraquara Departamento de Materiais Dentários e Prótese Araraquara São Paulo Brasil Universidade Estadual Paulista – UNESP, Faculdade de Odontologia de Araraquara, Departamento de Materiais Dentários e Prótese, Araraquara, São Paulo, Brasil.; 4 Universidade Estadual Paulista Universidade Estadual Paulista Faculdade de Odontologia de Araraquara Departamento de Fisiologia e Patologia, Araraquara São Paulo Brasil Universidade Estadual Paulista – UNESP, Faculdade de Odontologia de Araraquara, Departamento de Fisiologia e Patologia, Araraquara, São Paulo, Brasil.; 5 Universidade Estadual Paulista Universidade Estadual Paulista Faculdade de Odontologia de Araraquara Departamento de Clínica Infantil Araraquara São Paulo Brasil Universidade Estadual Paulista – UNESP, Faculdade de Odontologia de Araraquara, Departamento de Clínica Infantil, Araraquara, São Paulo, Brasil.

**Keywords:** Tooth bleaching, Dental pulp, Cytotoxicity, Odontoblasts

## Abstract

**Objective:**

This study was designed for the chemical activation of a 35% hydrogen peroxide (H_2_O_2_) bleaching gel to increase its whitening effectiveness and reduce its toxicity.

**Methodology:**

First, the bleaching gel - associated or not with ferrous sulfate (FS), manganese chloride (MC), peroxidase (PR), or catalase (CT) - was applied (3x 15 min) to enamel/dentin discs adapted to artificial pulp chambers. Then, odontoblast-like MDPC-23 cells were exposed for 1 h to the extracts (culture medium + components released from the product), for the assessment of viability (MTT assay) and oxidative stress (H_2_DCFDA). Residual H2O2 and bleaching effectiveness (DE) were also evaluated. Data were analyzed with one-way ANOVA complemented with Tukey’s test (n=8. p<0.05).

**Results:**

All chemically activated groups minimized MDPC-23 oxidative stress generation; however, significantly higher cell viability was detected for MC, PR, and CT than for plain 35% H2O2 gel. Nevertheless, FS, MC, PR, and CT reduced the amount of residual H2O2 and increased bleaching effectiveness.

**Conclusion:**

Chemical activation of 35% H2O2 gel with MC, PR, and CT minimized residual H2O2 and pulp cell toxicity; but PR duplicated the whitening potential of the bleaching gel after a single 45-minute session.

## Introduction

Hydrogen peroxide (H_2_O_2_) is found in high concentrations in bleaching gels widely used for in-office tooth-bleaching therapies.^1^ Whitening outcome is believed to be a consequence of H_2_O_2_ decomposition, which generates free radicals that interact with chromophores present in dentin substrate.^2^ Instead of being considered a strong oxidant agent, H_2_O_2_ is the reactive oxygen species (ROS) with the lowest oxidative potential^3^ . Therefore, to achieve effective dental color alteration in short periods, highly concentrated H_2_O_2_ bleaching gels (35-40%) have been traditionally used for professional tooth-bleaching.^1^ However, many studies have shown that such esthetic therapy allows diffusion of high amounts of H_2_O_2_ through enamel and dentin,^4 , 5^ causing *in vitro* ^6 - 11^ and *in vivo* ^12 - 15^ toxicity to pulp cells. Therefore, the “non-reacted H_2_O_2_” has been considered the main pathway for bleaching-induced tooth sensitivity, claimed by 80-100% of patients undergoing professional tooth-bleaching performed with high-concentrated gels.^16 , 17^

The cell toxicity mechanism mediated by bleaching gels on pulp cells has been correlated with two main pathways: (1) H_2_O_2_ arising in the pulp chamber during the bleaching procedure can diffuse through the cell membrane, followed by dissociation into free radicals on cytoplasm, causing a pathologic stress oxidative condition, lipid peroxidation, and necrosis; and (2) free radicals in the extracellular environment after H_2_O_2_ dissociation cause direct damage to cell membranes, leading to cell death by necrosis.^18^ The establishment of an inflammatory reaction and necrotic areas in pulp tissue of teeth subjected to professional bleaching has been observed in several *in vivo* studies.^12 - 15 , 19 - 21^ Recent demonstrations show H_2_O_2_ from bleaching gels induces pro-inflammatory cytokine release by pulp cells, negatively influencing their long-term regenerative potential.^8 , 11 , 14 , 15 , 22^

Therefore, many alternative therapies have been proposed to increase the biocompatibility of in-office bleaching therapy with the pulp-dentin complex, mainly aimed at reducing the amount of H_2_O_2_ capable of reaching pulp cells.^18^ Several authors have reported that reducing the H_2_O_2_ concentration in bleaching gels and the period of contact of these dental products with enamel may decrease the *in vitro* and *in vivo* toxicity caused by these esthetic therapies.^7 - 10 , 12 - 15 , 22^ However, additional sessions are needed to achieve bleaching outcomes similar to those of traditional therapies.^7 , 10 , 23^ Increasing the degradation rate of H_2_O_2_ has also been proposed as an alternative to minimizing residual H_2_O_2_ diffusion through enamel and dentin.^24 - 29^ Indeed, when manganese and iron-rich molecules are used to induce H_2_O_2_ decomposition into free radicals with Fenton and Fenton-like reactions, the H_2_O_2_ amount that reaches the pulp chamber *in vitro* is intensely reduced^24 , 27^ . Duque, et al.^29^ (2014) also demonstrated that association of 35% H_2_O_2_ with ferrous sulfate enhanced tooth-bleaching effectiveness and reduced H_2_O_2_ diffusion through enamel and dentin; but no significant minimization of odontoblast-like cell cytotoxicity was detected. Other studies have reported that plant extracts containing catalase and peroxidase enzymes, which can induce H_2_O_2_ degradation into free radicals, also have the potential to promote positive effects on bleaching effectiveness.^25 , 27^

Nevertheless, none of these studies compared the biological and esthetic effects of different substances capable of enhancing free radical release from H_2_O_2_ to select those that can be used as chemical activators of tooth-bleaching gels. Therefore, the aim was to evaluate the potential of Fenton’s reagents (ferrous sulfate and manganese chloride) and purified oxidoreductase enzymes (peroxidase and catalase) as chemical activators of a 35% H_2_O_2_ bleaching gel, on the trans-enamel/trans-dentinal diffusion of H_2_O_2_, pulp cell cytotoxicity, and bleaching effectiveness. Null hypothesis is that chemical activation has no effect on bleaching effectiveness, diffusion of residual H_2_O_2_, oxidative stress generation and cell viability reduction mediated by the 35% H_2_O_2_ bleaching gel on odontoblast-like cells.

## Methodology

### Enamel/dentin discs

Enamel/dentin discs (from 24- to 30-month-old bullocks), measuring 5.6 mm in diameter and 3.5 mm in thickness, were obtained as previously described.^29^ Enamel surface was cleaned with pumice stone solution under low speed handpiece (Dabi Atlante, Ribeirão Preto, SP, Brazil), and then evaluated with stereoscopic magnifying glass (Olympus 5ZX&, Olympus, São Paulo, SP, Brazil) to eliminate those samples with enamel defects, such as the presence of superficial cracks or hypoplasia. Dentin surfaces were cleaned with 0.5 M ethylenediaminetetraacetic acid solution (EDTA; Sigma-Aldrich, St. Louis, MO, USA), pH 7.2, for 30 s, to remove the smear layer, and the enamel surfaces were cleaned with a solution of pumice stone and distilled water at low speed.

### Bleaching procedure

After selecting the discs with sound enamel and standardized thickness at 3.5 mm, they were randomly distributed into the following groups: NC (negative control) – no treatment was performed on enamel; HP - 35% H_2_O_2_ gel (Whiteness HP 35%; FGM, Joinville, SC, Brazil); HP+FS - 35% H_2_O_2_ gel (Whiteness HP 35%; FGM) associated with ferrous sulfate (FeSO_4_·7H_2_O, chunks, ≥99% trace metals basis, Sigma-Aldrich); HP+MC - 35% H_2_O_2_ gel (Whiteness HP 35%; FGM) associated with manganese chloride (MnCl_2_, powder and chunks, ≥99% trace metals basis, Sigma-Aldrich); HP+PR - 35% H_2_O_2_ gel (Whiteness HP 35%; FGM) associated with horseradish peroxidase enzyme (type VI-A, lyophilized powder, 950-2000 units/mg solid, Sigma-Aldrich); and HP+CT - 35% H_2_O_2_ gel (Whiteness HP 35%; FGM) associated with catalase from bovine liver enzyme (powder, 2,000-5,000 units/mg protein, Sigma-Aldrich). For the chemically activated groups (HP+FS, HP+MC, HP+PR, and HP+CT), 1 mg of activators was incorporated into one drop of thickening agent (50 mL; Whiteness HP 35%; FGM) by manual mixing.^24 - 26 , 29^ Then, three drops of the H_2_O_2_ liquid phase (100 mL) were added, and the product was mixed for 15 s. This procedure was performed immediately before each application of the product. In the HP group, the same procedure was performed but no chemical substance was incorporated. The manipulation of the bleaching gel (1 drop of thickening per 3 drops of H_2_O_2_) was based on the manufacturer’s instructions (FGM). In total, three 15-minute applications were performed in all bleached groups, and a 40-mL volume of bleaching gels was applied to enamel at each 15-minute application, via a pipette coupled with a capillary piston tip (Microman E, Gilson, Middelton, WI, USA), which allows standardized pipetting of viscous liquid. Digital images were taken at a 45 cm distance from the specimens, with a DSLR camera (Nikon D3300; F 22, ISO 200, speed 180; Melville, NY, USA) coupled with macro lenses (90 mm; F2.8. Tamron, Colonia, Italy) and a circular flash (EM-140DG Macro flash, speed 1/16; Sigma Corporation of America, Ronkonkoma, NY, USA) to observe the reaction of the bleaching gel with the chemical activators during the 15-minute application time. The pH was measured with a bench top pH meter (HI2221 Calibration Check pH/ORP Meter, Hanna Instruments, Woonsocket, Rhode Island, NE, USA), coupled with a microprobe (HI1131P General Purpose pH Electrode, Hanna Instruments). The bleaching gels were manipulated in a 1.5 mL tube, and the probe was submersed into the material. The pH was checked after 0.5, 5, 10, and 15 min. A total of 4 samples per group was manipulated for this analysis.

### Trans-enamel and trans-dentinal cytotoxicity

Enamel/dentin discs were adapted to artificial pulp chambers (APC), as previously described.^29^ The APC/disc sets (sterilized in ethylene oxide) were placed in 24-well plates with dentin in contact with 1 mL of DMEM (Dulbecco’s Modified Eagle Medium; supplemented with 100 IU/mL penicillin, 100 mg/mL streptomycin, and 2 mmol/L glutamine; Gibco, Grand Island, NY, USA). Enamel remained exposed to receive the treatments according to each group. Immediately after the bleaching procedure, the culture medium (extract) was collected and distributed into 100-mL aliquots, which were applied for 1 h to odontoblast-like MDPC-23 cells previously seeded in an 80% confluence pattern [1x10^4^ cells/96-well plates in DMEM plus 10% fetal bovine serum (FBS), for 24 h at 37°C and 5% CO_2_]. Cell viability was assessed by incubation of the cells for 4 h in DMEM without FBS (Gibco) supplemented (10:1) with 5 mg/mL MTT solution (Sigma-Aldrich) at 37°C and 5% CO_2._ The absorbance of formazan crystals in the viable cells was read (570 nm; Synergy H1, BioTek, Winooski, VT, USA), and the mean absorbance of the NC group was considered 100% of cell viability (n=8). Oxidative stress was evaluated in cells pre-treated with fluorescence probe carboxy-H_2_DCFDA (5 mM; Invitrogen, Carlsbad, CA, USA) (n=8), and then exposed for 1 h to the extracts. After this period, the fluorescence intensity was monitored at 59 nm excitation and 517 nm emission (Synergy H1, BioTek), and the fold increases were calculated after normalization with the NC group.

### Quantification of residual H2O2

A 100-µL aliquot of the extract collected from the same samples of trans-enamel and trans-dentinal cytotoxicity assay (n=8) was transferred to tubes containing 900 mL of acetate buffer solution (2 mol/L, pH 4.5), to avoid H_2_O_2_ degradation. Then, 500-µL of buffer solution plus extract was transferred to experimental tubes to react with leuco crystal violet (0.5 mg/mL; Sigma-Aldrich) and horseradish peroxidase enzyme (1 mg/mL; Sigma-Aldrich). The final volume of reaction was adjusted to 3 mL with distilled water, and the optical density of the solutions was measured at 600 nm wavelength (Synergy H1, BioTek). To estimate the effects of chemical activators on H_2_O_2_ diffusion, the HP group was considered 100% of H_2_O_2_ diffusion, and diffusion percentages for chemically activated groups were calculated based on this parameter.

### Color alteration measurement

For this analysis, the discs were subjected to staining with black tea to standardize the baseline color (n=8).^29^ This procedure was performed to randomly distribute specimens with similar L* and b* values (CIE L*a*b* system) among the control and experimental groups, obtaining standardized samples. Darkened samples were used to verify the potential of the different formulations to fasten the whitening outcome compared with the gel with no chemical supplementation.

Specimens were kept in 100% humidity to standardize the hydration pattern. For that, the discs were placed at the bottom of wells of 24-well plates in a way that dentin was maintained in contact with a cotton pellet embedded with deionized water, and the enamel was covered with a cotton pellet embedded in artificial saliva solution.^10 , 23^ The plates were kept in incubator for 72 h, at 37°C (Orion 515, Fanen, São Paulo, SP, Brazil). A color readout was then used to obtain baseline (BS) values for each disc. To be read, the discs were positioned in a white silicone matrix, leaving only the enamel surface exposed. A portable UV-VIS spectrophotometer with 4 mm aperture (Color Guide 45/0; BYK-Gardner GmbH, Geretsried, BAV, Germany) was positioned over the center of the 5.6 mm diameter disc, so that only the surface of the sample was read with no interference of the background color. The samples were read in sequence by the same operator. A total of three sequential readings was performed to eliminate bias from spectrophotometer positioning onto the sample.

Bleaching protocol was performed on enamel surfaces, after each enamel was washed with deionized water and dried with filter paper. The specimens were incubated in 100% humidity for 72 h to allow for rehydration, and a post-bleaching (PS) color readout was obtained. Values of L* a* b* were recorded to obtain ΔL, Δa, and Δb values for each sample by the following equation: Δ(L, a or b) = PS value (L*, a*, or b*) – BS value (L*, a*, or b*). Overall color change of each specimen, expressed as ΔE, was calculated according to the equation ΔE=[(ΔL)^2^ +(Δa)^2^+(Δb)^2^]½.

### Statistical analysis

Sample size was calculated with DDS Research (Sample Size Calculator, average, two samples, a=5%; b=95%), and eight samples per group were stablished for each assay. Percentage of cell viability (MTT assay) (NC *versus* HP) and DE (NC *versus* HP) were used as parameters. Power calculation analyses were also performed by DDS Research (Statistical Power Calculator, average, two sample, two-tail test, a=5%) at the end of the experiment, showing 100% statistical power for each evaluation. Two independent experiments were performed for each assay. Data were compiled and analyzed by Kolmogorov-Smirnov and Levene tests. Since normal data were obtained, one-way ANOVA and Tukey’s test were used for cell viability, as well as H_2_O_2_ diffusion, oxidative stress, DE, DL, Da, and Db analysis. Data of pH measurement were analyzed with repeated measure two-way ANOVA and Dunnet’s test to compare pH values at each time-point with those from HP group. All statistical analyses were carried out at a significance level of 5%.

## Results

### Chemical activation characteristics

**Figure 1:**
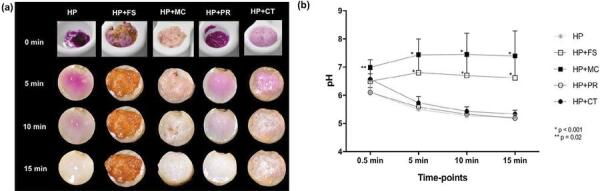
(a) Representative digital images of bleaching gel reaction in the presence or not of chemical activators, immediately after manipulation (0 min) and after 5, 10, and 15 min on enamel/dentin discs. Note the bubbles in HP+FS, HP+MC, and HP+CT groups. Deposition of brown sub-products can be observed in groups HP+FS and HP+MC. (b) Graph representative of mean (standard deviation) values of pH for the bleaching gels at 0.5, 5, 10, and 15 min. Asterisks indicates significant difference with the HP group (repeated measures Two-way ANOVA/Dunnet’s test, p<0.05)

Reaction immediately before manipulation and after 5, 10, and 15 minutes on tooth surfaces can be observed in Figure 1a . Bubbles were observed in all chemically activated groups and deposition of brown sub-products was detected only in groups HP+MC and HP+FS. pH values of the bleaching gels at each time-point and the statistical analysis through time can be observed in Figure 1b . The statistical analysis through time can be observed in Figure 1b . Significant increase in the pH compared with HP group was detected for HP+FS at 5 min and for HP+MC at 0.5 min. The enzymes peroxidase and catalase had insignificant influence on the pH of the 35% H_2_O_2_ gel.

### Biologic assays

**Figure 2:**
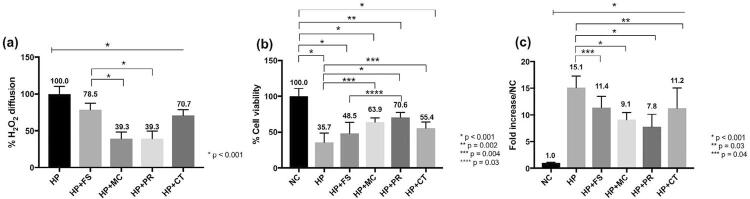
(a), (b), and (c) Bar graph of mean values (standard deviation) of cell viability percentages (MTT assay), fold increase in carboxy-H2DCFDA fluorescence (oxidative stress), and residual H2O2 percentages (violet leuco-crystal/peroxidase assay), respectively. Numbers are mean values for each assay. demonstrates the comparison between groups from the start to end points under the line; demonstrates the comparison between groups; asterisk indicates significant difference (one-way ANOVA/Tukey’s test; p<0.05)

Considerable cell viability reduction was observed for all bleached groups compared with NC; however, cell viability higher than that of the HP group was determined for the HP+MC, HP+PR, and HP+CT groups, with HP+PR featuring the greatest value ( Figure 2a ). Increased oxidative stress was detected for all groups related to NC; but all bleached groups associated with chemical activators featured oxidative stress substantially lower than that found in the HP group ( Figure 2b ).

### Residual H2O2 quantification

Reductions in H_2_O_2_ diffusion occurred for those extracts obtained from bleached groups associated with chemical activators compared with plain H_2_O_2_ gel (HP group). The HP+MC and HP+PR groups featured significantly lower H_2_O_2_ amounts on extracts compared with the other groups ( Figure 2c ).

### Bleaching effectiveness

**Figure 3:**
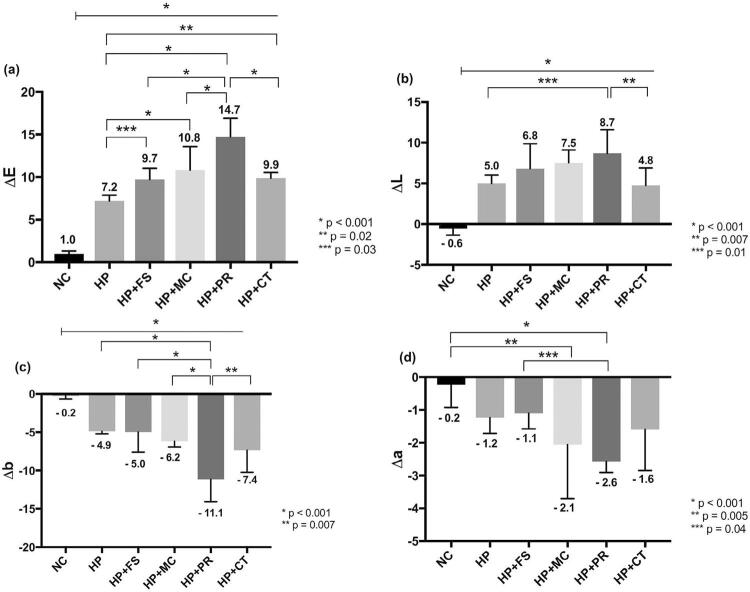
(a), (b), (c) and (d) Bar graph of mean (standard deviation) of DE, DL, Db, and Da values, respectively, for color alteration assay (CIE L*a*b*). Numbers are mean delta for each group. demonstrates the comparison between groups from the start to end points under the line; demonstrates the comparison between groups; asterisk indicates significant difference (one-way ANOVA/Tukey’s test; p<0.05)

All bleached groups presented notably higher DE values than the NC group. HP+PR featured the highest DE value, which was significantly different from that of the other groups ( Figure 3a ). All the bleached groups promoted significant increase in DL and decrease in Db compared with NC group; nevertheless, only HP+PR featured significant differences with HP group for both parameters. Considerable reduction in Da related to NC group was detected for HP+PR and HP+MC groups.

## Discussion

Catalyzed H_2_O_2_ propagation has been used to increase the oxidative potential of H_2_O_2_, that, in turn, eliminates organic contaminants from aquifers and soil^3^ . Depending on environmental conditions, H_2_O_2_ may be dissociated into different molecules, releasing oxygen (O_2_), water (H_2_O), and highly reactive free radicals, such as hydroxyl (HO^·^), peri-hydroxyl (HO_2_^·^), and superoxide (O_2_^·^) radicals.^30^ Following the same catalytic mechanism, the aim was to reduce the amount of residual H_2_O_2_ on tooth-structure and consequently the cytotoxicity with pulp cells, by increasing the reactivity of the product with dental structure after incorporating ferrous sulfate, manganese chloride, peroxidase, or catalase into a highly concentrated bleaching gel. We chose a 35% H_2_O_2_ gel to evaluate the most challenging situation, since the indirect cytotoxicity of such dental products has been well-demonstrated in literature.^6 - 10^ The bleaching effectiveness was also assessed to demonstrate whether the chemical activators indeed increase the interaction of H_2_O_2_ with mineralized dental tissue. The null hypothesis of this study was partially rejected, since all chemical substances enhanced the esthetic outcomes, reduced residual H_2_O_2_ diffusion and oxidative stress generation compared with the HP group. However, cell viability reduction mediated by the 35% H_2_O_2_ gel (HP group) was only significantly reduced by manganese chloride, peroxidase, and catalase.

**Figure 4:**
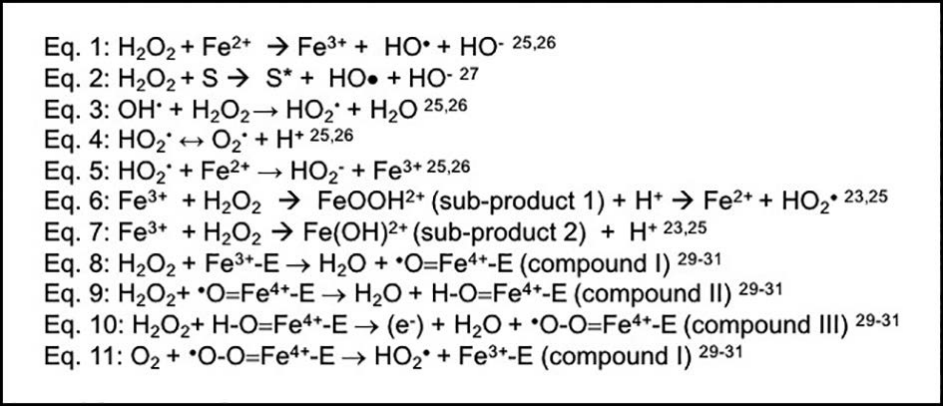
Degradation of H2O2 in the presence of chemical activators. Eq.=equation. S=substrate. S* oxidized substrate. E=enzyme

Ferrous sulfate and manganese chloride were used to induce H_2_O_2_ catalysis with Fenton and Fenton-like reactions, respectively, in which the decomposition of H_2_O_2_ generates hydroxyl radicals (HO^·^),^31 - 33^ as shown in Figure 4 (Eq.1 and 2). In the presence of high concentrations of H_2_O_2_, HO^·^ interacts with excess H_2_O_2_ to promote a series of propagation reactions ( Figure 4 , Eq. 3-5), resulting in the release of other free radicals, such as HO_2_^•^ and O_2_^•^.^31 , 32^

According to our results, ferrous sulfate reduced residual H_2_O_2_ diffusion by 21.5%; yet it had insignificant effect on cell viability compared with HP group. On the other hand, manganese chloride reduced the residual H_2_O_2_ diffusion across enamel and dentin by 60.7%, associated with substantial minimization in cell viability reduction promoted by HP group. These effects were related with increased reduction in oxidative stress generation by HP+MC group, compared with HP+FS. Both manganese chloride and ferrous sulfate enhanced considerably the DE values compared with plain 35% H_2_O_2_ gel. This result was corroborated by Duque, et al.^29^ (2014), who also observed adding ferrous sulfate to a commercial bleaching gel containing 35% H_2_O_2_ enhanced the bleaching effectiveness; however, only an insignificant reduction in the trans-enamel and trans-dentinal cytotoxicity was observed.^29^ As shown in Figure 1 , this chemical activator caused brown sub-product deposition, leading to H_2_O_2_ consumption with no release of free radicals, minimizing the effectiveness of the reaction.^3 , 30^ Reactions related to this process are demonstrated in Figure 4 (Eq. 6 and 7). Therefore, formation of these sub-products may be considered a disadvantage of the iron-based Fenton reaction.

We also observed a slight deposition of brown sub-products during manganese chloride reaction with the bleaching gel, which disappeared at the end of the reaction ( Figure 1 ). According to the literature, soluble manganese is a stoichiometrically efficient catalyst for the generation of hydroxyl radicals; however, depending on pH regime, an amorphous manganese oxide precipitate occurs.^33^ Nevertheless, this component is capable of catalyzing H_2_O_2_ to release O_2_^•^, HO_2_^•^, and H^27^ . The advantage of using manganese instead of iron is based on the high redox potential of manganese, which makes this molecule more prone to react with H_2_O_2_.^32 , 33^ Differently from iron, which dissociates into intermediates for electron exchanges during reaction with H_2_O_2_, manganese, in turn, makes the reaction more effective ( Figure 4 , Eq. 7).^32^ In previous studies, researchers demonstrated that manganese gluconate/chloride increases the bleaching efficacy of H_2_O_2_ by around 1.5-8 times.^24 - 26^ Additionally, it was shown that the concentration of H_2_O_2_ and the time for diffusion through enamel and dentin are significantly reduced in the presence of manganese-containing activators,^24 , 28^ such as it was observed in this investigation.

Addition of peroxidase or catalase to the 35% H_2_O_2_ gel also had a positive effect on biologic and esthetic features. Both enzymes minimized the cell viability reduction, oxidative stress generation, and residual H_2_O_2_ diffusion, as well as increased the DE values compared with those of the HP control group. These molecules comprise a group of enzymes called oxidoreductases, which contain an active metallic center in a Fe^3^resting state. They are found on living tissues and act to scavenge the H_2_O_2_ arising from the respiratory chain and oxidative stress.^34^ Conversion of H_2_O_2_ into H_2_O and O_2_ by these enzymes involves two well-understood one-electron reduction steps, called peroxidase cycle. The Fe^3^ metallic center of catalase and peroxidase reduces one molecule of H_2_O_2_ into H_2_O, generating a covalent Fe^4^O oxyferryl species, referred to as compound I, which is two oxidizing equivalents above the resting state. When this intermediate reaction oxidizes a second H_2_O_2_ molecule, O_2_ and H_2_O are formed, generating a second intermediate enzyme, compound II, which is one oxidizing equivalent above the resting state. The second one-electron reduction step returns compound II to the resting state of the enzyme. Both compounds I and II are powerful oxidants, with redox potentials estimated to be close to +1 V^35 - 37^ ( Figure 4 , Eq. 8 and 9). An oxidase cycle has been demonstrated by Berglund, et al.^36^ (2002) for horseradish peroxidase in the presence of excess H_2_O_2_. According to the author, compound II may be transformed into several oxidative species when in contact with H_2_O_2_, leading to the release of HO_2_^·^ species ( Figure 4 , Eq. 10 and 11). Therefore, the suggestion is that peroxidase and catalase induced the formation of highly oxidative intermediates when added to the 35% H_2_O_2_ bleaching gel, increasing the oxidative potential of the product. Gopinath, et al.^27^ (2013) demonstrated that 10% and 35% H_2_O_2_ gels reached the same bleaching outcome in the presence of a natural extract rich in catalase and peroxidase, regardless of the H_2_O_2_ concentration. Travassos, et al.^25^ (2010) observed a 42.1% increase in bleaching effectiveness (DE) for a 35% H_2_O_2_ gel after one single 3x10-minute tooth-whitening session when a peroxidase-rich extract was incorporated into the dental product. The authors speculated that this positive effect was related to the enhanced free radical formation mediated by this enzyme.

Overall, the indication is that the release of free radicals mediated by the interaction of H_2_O_2_ with chemical activators increase bleaching effectiveness, minimizing the amount of residual H_2_O_2_ that diffuse through enamel/dentin to cause toxic effects to the cultured pulp cells. However, one important limitation of this study is that free radicals released from the bleaching gels and their trans-enamel and trans-dentinal diffusion were ignored. Therefore, the cytotoxicity observed here for all chemically activated groups may also be related to the release of such reactive molecules capable of diffusing through enamel/dentin to reach the cells. This effect may be related to the contradictory results found for H_2_O_2_ diffusion on HP+MC group. Instead of achieving the lowest H_2_O_2_ diffusion, this group had the same behavior as HP+FS and HP+CT groups for all the other parameters tested. We believe that manganese chloride increases the formation of free radicals in comparison with ferrous sulfate and catalase, but these free radicals may have diffused through enamel and dentin to play a role in the cytotoxicity to the pulp cells.

On the other hand, peroxidase also had the lowest amount of residual H_2_O_2_, which was statistically similar to manganese chloride. Nevertheless, the better cell parameters and whitening outcome found for HP+PR group compared with HP+MC may result from the oxidase cycle reaction, since the reactive species described as compound I and III may have played a role in the reaction of H_2_O_2_ with tooth structure, resulting in a lower amount of free radical diffusion through enamel/dentin discs. Therefore, more studies are needed to demonstrate the pathway related to the minimization of the toxicity mediated by the chemical activators tested here. Non-stained discs were used in the biological assays to avoid the release of toxic components of black tea to the cells, as performed before.^7 , 10 , 23 , 29^ Since free radicals released by the bleaching gel were expected to react with the chromophores, we may speculate that a high amount of non-reacted molecules may have diffused to cause the toxic effects observed in this investigation. Also, several other relevant biological factors found *in vivo* , such as the presence of pulp pressure, extracellular matrix, and host immune cells, have been rejected in laboratorial tests to assess the biological and esthetic behavior of dental products.^18^ Hence, the results found in this *in vitro* study can be considered overestimated.

Torres, et al.^28^ (2013) reported that the use of chemical activators to minimize the residual H_2_O_2_ diffusion to the pulp chamber seems to be more evident when low-concentration bleaching gels are used. According to those authors, 35% H_2_O_2_ formulations provide greater availability of residual H_2_O_2_, so, likely, the chemical activator is unable to reduce the rate of peroxide diffusion at secure levels. The literature has already shown that high-concentrated bleaching gels have intense potential to bleach teeth from the first 45-minute session.^16 , 17^ Nevertheless, the increase in bleaching effectiveness demonstrated in this study may be useful to fasten the whitening outcome on dark-colored teeth, such as tetracycline stained teeth; however, more studies are needed to prove the effectiveness of the formulations tested in these specific situations.

The focus of the current studies is to reduce the concentration of in-office bleaching gels to minimize the negative effects on pulp cells.^38 , 39^ It has been previously demonstrated that a 50% reduction in the concentration of peroxide in bleaching gels with 35% H_2_O_2_ results in a significant positive effect on cell viability; but the bleaching effectiveness is harmed.^7 , 23^ Therefore, since the manganese chloride, catalase, and peroxidase were capable of minimizing the cytotoxicity of a highly concentrated bleaching gel by increasing its reactivity with tooth structure, it seems reasonable that they may be considered a promising alternative to improve the biocompatibility and esthetic outcomes of low-concentrated whitening products. However, considering the limitations of this *in vitro* study, further investigations are needed to clarify the benefits of chemical activations to commercial bleaching agents and their safety for clinical applications. Also, a cost-benefit analysis should be performed, since the purified horseradish peroxidase enzyme used here has a high cost. Evaluating other sources of this enzyme and performing dose-response studies may clarify the benefits of this substance as a chemical activator of tooth bleaching products.

## Conclusion

Chemical activation of 35% H_2_O_2_ bleaching gel with manganese chloride, peroxidase, or catalase enhances tooth-whitening and reduces the amount of residual H_2_O_2_ capable of diffusing through enamel and dentin, minimizing the toxic effects of this product to pulp cells. Enzymatic activation with peroxidase featured the best biological and esthetic results compared with those of the other chemical activators.
